# Commentary: Plastid establishment did not require a chlamydial partner

**DOI:** 10.3389/fcimb.2016.00043

**Published:** 2016-04-13

**Authors:** Steven G. Ball, Debashish Bhattacharya, Huan Qiu, Andreas P. M. Weber

**Affiliations:** ^1^Unité de Glycobiologie Structurale et Fonctionnelle, UMR8576 Centre National de la Recherche Scientifique-Université des Sciences et Technologies de LilleVilleneuve d'Ascq, France; ^2^Department of Ecology, Evolution and Natural Resources, Rutgers, The State University of New Jersey, New Brunswick, NJ, USA; ^3^Center of Excellence on Plant Sciences, Institute for Plant Biochemistry, Heinrich-Heine-UniversityDüsseldorf, Germany

**Keywords:** endosymbiosis, mitochondria evolution, plastid evolution, eukaryote evolution, Chlamydiales, Rickettsiales

Several groups have independently proposed an active role for Chlamydiales in primary plastid establishment in Archaeplastida (Huang and Gogarten, 2007; Becker et al., 2008; Moustafa et al., 2008). We relied on a combination of biochemical and phylogenetic evidence to erect the MAT (Ménage à Trois) hypothesis (Ball et al., 2013; Facchinelli et al., 2013). Under this scenario, Chlamydiales sheltered the once free-living cyanobacterial plastid ancestor from host defenses and provided critical components such as carbohydrate transporters and protein effectors that allowed the storage of exported carbohydrates into host glycogen pools. A recent paper by Domman et al. (2015) reassessed the phylogenies published by us and others on these components. These authors applied evolutionary models that better account for across-site and across-branch sequence compositional variation (i.e., Bayesian approaches with the CAT family of evolutionary models Lartillot and Philippe, 2004) to reanalyze proteins involved in glycogen metabolism. These are either chlamydial effectors (GlgC, ADP-glucose pyrophosphorylase; GlgP, glycogen phosphorylase; GlgX, glycogen debranching enzyme; and GlgA, glycogen synthase) or chlamydial transporters (UhpC, G6P import protein). Previous trees often used automated phylogenomic pipelines that relied on single-matrix (usually best-fit) substitution models (e.g., LG, WAG) that could potentially provide incorrect inference due to rate heterogeneity across sites (Morgan et al., 2013). Based on their results, the authors (Domman et al., 2015) argued that GlgC and GlgP now show evidence of being of cyanobacterial and host origin, respectively, that Chlamydiales and Archaeplastida are united by the LGT (Lateral Gene Transfer) of GlgX but that the direction of transfer from chlamydiales to Archaeplastida is no longer clear. Furthermore, our hypothesis that chlamydiales have provided GlgA and UhpC to the Archaeplastida is now in question. Below, we inspect these issues in detail.

## Glgc and glgp

Domman et al. (2015) favor a cyanobacterial and host origin, respectively, for ADP-glucose pyrophosphorylase and glycogen phosphorylase in Archaeplastida with no involvement of Chlamydiales. We fully agree with this hypothesis and have presented it on several occasions (reviewed in Ball et al., 2011). The problem we see here is that by including these genes in their study with the aim of rejecting the MAT hypothesis, these authors imply that because GlgC and GlgP are chlamydial effector enzymes, they should also have been chlamydial LGTs in extant Archaeplastida. This is incorrect and we have stated the opposite (Ball et al., 2013). To clarify, among the chlamydial effectors, only GlgX and GlgA were needed to establish the initial symbiosis.

## Glgx

**Figure 1:**
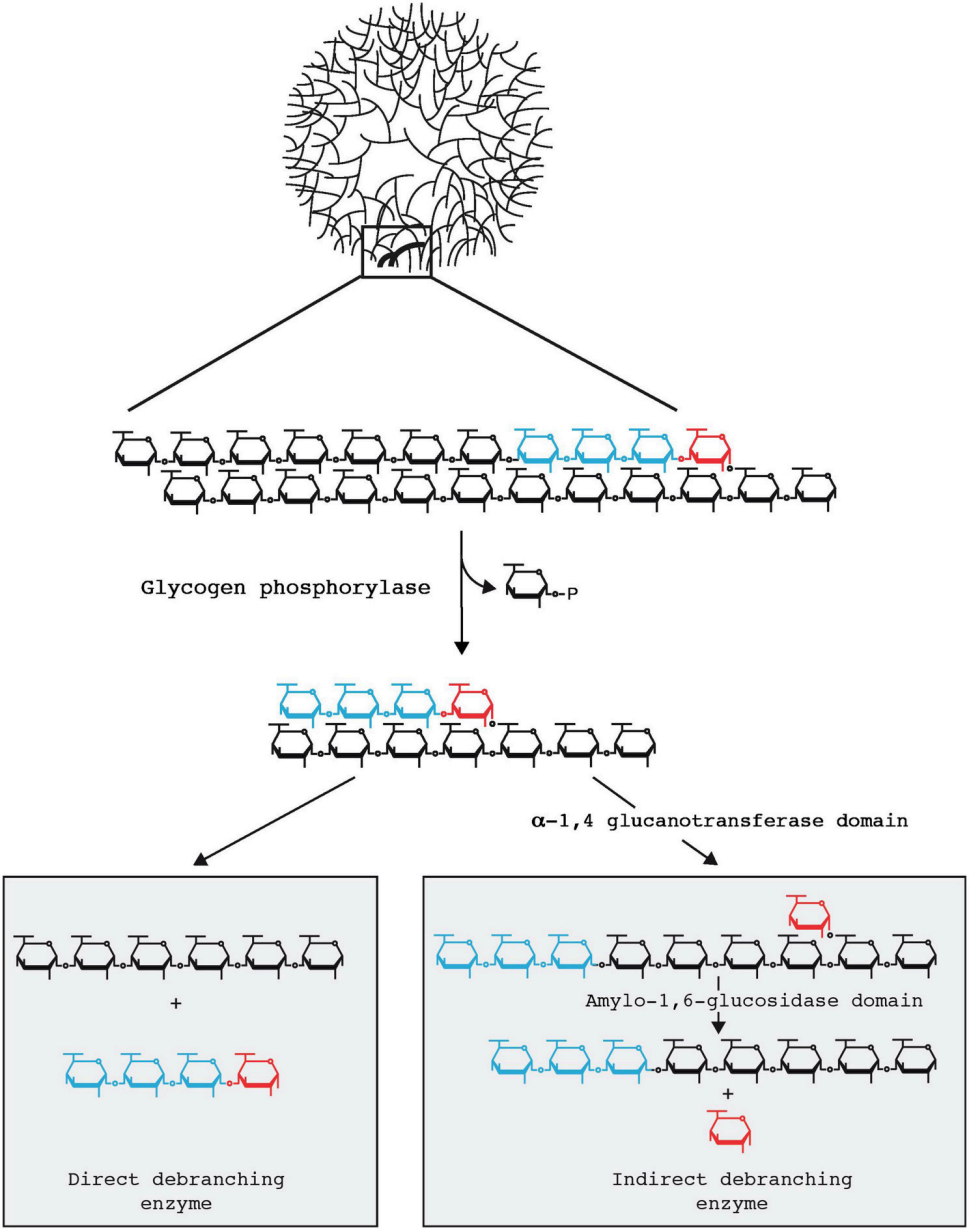
**Glycogen particle debranching in eukaryotes and bacteria**. A 40 nm diameter hydrosoluble glycogen particle is displayed on top emphasizing the outer chains connected to the particle through a single α-1,6 linkage. One-third in weight of the total glucose resides are these outer chains which are directly available to the hydrosoluble enzymes involved in glycogen breakdown. In both bacteria and eukaryotes these outer chains enlarged in the top panel are first recessed through glycogen phosphorylase which in the presence of orthophosphate releases glucose-1-P. All reported glycogen phosphorylases stop at a four glucose residue distance from the first encountered α-1,6 branch (displayed in red). Bacteria and eukaryotes differ at the subsequent steps (Cenci et al., 2014). Bacteria directly hydrolyse the remaining outer α-1,6 linkage through the so-called direct debranching enzyme (DBE) generating a smaller glycogen particle and releasing a small chain of four glucose residues (maltotetraose) in the cytosol. Bacteria must then metabolize these small chains and do so with the help of MOS metabolism enzymes. DBE belongs to the GH13 glycosyl hydrolase family. Eukaryotes first hydrolyse the α-1,4 preceding the branch and transfer the maltotriose (displayed in blue in the right panel) outer chain to the neighboring chain allowing for further digestion with glycogen phosphorylase. The α-1,6 linked glucose at the remaining branch is then hydrolyzed by a second active site on the same enzyme named indirect debranching enzyme (iDBE) releasing glucose which is phosphorylated to glucose-6-P by hexokinase. No MOS is released in the cytosol of eukaryotes through glycogen metabolism. iDBE is derived from a gene fusion between a GH13 and GH133 domain. Consequently one finds iDBE candidate sequences in all eukaryotes unrelated to Archaeplastida that accumulate glycogen [all opisthokonts (fungi and animals), amoebozoa, alveolata, glycogen accumulating excavata (*Trichomonas, Giardia*)] whereas the fusion has never been seen in bacteria despite the extensive databases that are available (Ball et al., 2011, 2015). Bacterial GH13 DBE is likewise never seen in the aforementioned eukaryotic clades unrelated to Archaeplastida. In addition eukaryotes do not contain cytosolic enzymes of MOS metabolism with the exception of maltase (DPE2) which selectively hydrolyses maltose generated by the eukaryotic specific enzyme β-amylase.

Domman et al. (2015) accept that Chlamydiales and Archaeplastida are united through LGT in this phylogeny. However, the direction of transfer is judged to be unclear due to the tree topology. Their phylogeny generally matches the one we have previously presented (Ball et al., 2013). We have emphasized previously that within the group of sequences proven to be united by LGT, phylogenetic signal erosion does not allow conclusions to be drawn about direction of transfer. Importantly, as described below, our hypothesis in this regard is not based solely on phylogenetic data but rather on what we consider to be compelling biochemical evidence. By this, we mean that GlgX is a direct debranching enzyme of bacterial origin, absent in eukaryotes (Cenci et al., 2014). This enzyme ties together glycogen and malto-oligosccharide (MOS) metabolism in the bacterial cytosol as depicted in Figure 1. Glycogen-accumulating heterotrophic eukaryotes use a different glucose-generating pathway under control of an indirect debranching enzyme with no release of MOS (Cenci et al., 2014). Eukaryotes typically lack cytosolic MOS metabolism while able occasionally to degrade maltose. Therefore, indirect and direct debranching defines eukaryotes and bacteria, respectively, which clearly distinguishes their glycogen and carbohydrate metabolisms. In summary, by combining their phylogeny, which supports LGT but is inconclusive with regard to the direction of transfer, with knowledge of biochemistry, we surmise that Archaeplastida gained the GlgX-encoding gene from Chlamydiales.

## Glga and uhpc

The analysis done by Domman et al. (2015) emphasizes the unclear origin of both genes within Archaeplastida. Here again, we have reported the same result and discussed this issue in detail (Ball et al., 2013). In both cases, whereas the Chlamydiales remain the closest branching taxa to the Archaeplastida root, we cannot exclude a small group of more distantly related diverse Proteobacteria as potential LGT donors to Archaeplastida (UhpC and GlgA). We have however not espoused the idea that phylogenetic data alone prove LGT from Chlamydiales to Archaeplastida, but strongly suggested it. This reflects the relatively close and proximal position of Chlamydiales with respect to the root of Archaeplastida and despite the ever-growing database of Proteobacteria the taxonomically poorly sampled Chlamydiales remain as the most closely related clade to algae and plants. Once again some inference with respect to biochemistry can be made in the face of obvious phylogenetic uncertainty. Both GT5 types of ADP-Glc specific glycogen (starch) synthases and UhpC-like proteins are not present in eukaryotes, with the exception of Archaeplastida. In addition, UhpC-like proteins are glucose-6-P sensors and do not function in glucose-6-P transport in free-living bacteria as found in Chlamydiales. This strongly suggests that the neofunctionalization of a hexose-phosphate transporter from a hexose-phosphate sensor (UhpC) occurred in an intracellular bacterium, likely Chlamydiales. Therefore, for both UhpC and GlgA, the direction of transfer is presumed to be from bacteria to Archaeplastida. Moreover, an uninterrupted diversity of cyanobacteria in this tree shows congruence with the 16S rRNA phylogeny. The node uniting these taxa is >2 billion years old and predates Archaeplastida diversification and plastid endosymbiosis. The possible alternative topology of the GlgA tree inferred by Domman et al. that rejects Chlamydiales as “donors” of the GlgA gene places the Archaeplastida at the root of this tree, implying that Archaeplastida diversification predates that of cyanobacteria, which is by all accounts, untenable.

In conclusion, in spite of the strong language used by Domman et al. their results shed no new light on primary plastid endosymbiosis and in fact do not differ significantly enough from our (and other) published works to overturn tripartite hypotheses. Therefore, we stand by the MAT hypothesis and the idea that intracellular bacteria were essential to mitochondrial and plastid acquisition (Ball et al., 2016). We will await novel data from natural Chlamydiales pathogens or the results of biochemical experiments that will, in our opinion, more likely tilt the argument in one direction or another.

## Author contributions

SB coordinated the writing and wrote this manuscript together with inputs from all other listed co-authors. All authors made equal contributions in finalizing this manuscript.

## Funding

SB was supported by the CNRS, the Université de Lille CNRS, and the ANR grants “expendo” and “ménage à trois.” DB was supported by NSF grants MGSP 0625440 and MCB 0946528, and AW was supported by German Research Foundation grants CRC-TR1, CRC 1208, and EXC 1028.

### Conflict of interest statement

The authors declare that the research was conducted in the absence of any commercial or financial relationships that could be construed as a potential conflict of interest. The reviewer JC and handling Editor declared a current collaboration and the handling Editor states that the process nevertheless met the standards of a fair and objective review.
